# A clarification of authorship in an old publication

**Published:** 2016-11-18

Recently, the editorial office of Zoological Research (ZR) received a statement from Prof. Shu-Yi Zhang regarding his authorship of an old publication (Ma et al., 2003) in ZR (see below). He requested to withdraw his authorship of this article. The editorial office took this statement seriously and the editor-in-chief and associate editors-in-chief had in-depth discussions on this issue. Despite the fact that ZR announces clearly that it copes with the authorship measurements of International Committee of Medical Journal Editors (ICMJE, 2013), and gives guidance to authors regarding the proper authorship in the Instruction to Author, the actual practice still depends on the author himself or herself (Liu, 2016). As a way to clarify the related concerns, ZR decided to publish the clarification provided by Prof. Zhang and the corresponding replies from the other co-authors of this article. 

Editorial Office of Zoological Research

**Statement from Dr. Shu-Yi Zhang**

I write to you regarding the research report entitled "Piscivorous Habit and Echolocation Sound of Myotis ricketti at Fangshan, Beijing" 2003, 24(4): 265-268, of which I'm listed as one of the two co-corresponding authors. When the manuscript was submitted from Beijing to Zoological Research, I was carrying out a long-term investigation on SARS outbreak in Southern China, and was not informed of the manuscript submission. I did not participate in the drafting of this manuscript; therefore, I would like to withdraw my authorship of this article. Otherwise, I will not bear any responsibilities of data or words in this research report. 

Shu-Yi Zhang

*szhang@syau.edu.cn*

College of Animal Science and Veterinary Medicine

*Shenyang Agricultural University*

**Replies from the other authors of this article**

The ZR editorial office sent Dr. Shu-Yi Zhang's letter to all authors of this article. Dr. Bing Liang (Institute of Zoology, Chinese Academy of Sciences) and Dr. Gareth Jones (University of Bristol, UK) expressed their support to Dr. Zhang's request of withdrawing his authorship. In his e-mail to ZR editorial office, Dr. Jones wrote "*Professor Zhang's request seems very reasonable to me, and I support it. Because the paper is in Chinese (which I cannot read), I was not aware of it either. I appreciate Dr, Ma's generosity in including me as an author, but because I was unaware of the paper, I should not be included as an author either*".

Dr. Jie Ma (Harvard University), the first author of this article, replied that "*I apologize to those authors who did not read the draft before the submission. Therefore, I would like to fully respect the authors' requests to remove their authorship from this paper*".

## References

[1] International Committee of Medical Journal Editors. 2013 Defining the Role of Authors and Contributors.

[2] Liu SQ. 2016 Who is innocent in authorship misconduct?. *Zoological Research*, 37 (3): 117- 118. 27265648 10.13918/j.issn.2095-8137.2016.3.117 PMC4914573

[3] Ma J, Zhang LB, Liang B, Shen JX, Zhang SY, Jones G. 2003 Piscivorous habit and echolocation sound of *Myotis ricketti* at Fangshan, Beijing. *Zoological Research*, 24 (4): 265- 268.

